# A novel, homozygous mutation in *desert hedgehog* (*DHH*) in a 46, XY patient with dysgenetic testes presenting with primary amenorrhoea: a case report

**DOI:** 10.1186/s13633-018-0056-3

**Published:** 2018-03-02

**Authors:** Karen M. Rothacker, Katie L. Ayers, Dave Tang, Kiranjit Joshi, Jocelyn A. van den Bergen, Gorjana Robevska, Naeem Samnakay, Lakshmi Nagarajan, Kate Francis, Andrew H. Sinclair, Catherine S. Choong

**Affiliations:** 1 Department of Endocrinology and Diabetes, Princess Margaret Hospital, Subiaco, WA Australia; 2 Murdoch Childrens Research Institute, Melbourne, VIC Australia; 3 Department of Paediatrics, The University of Melbourne, Melbourne, VIC Australia; 4 Telethon Kids Institute, Subiaco, WA Australia; 5 Department of Paediatric Surgery, Princess Margaret Hospital, Subiaco, WA Australia; 6 School of Surgery, The University of Western Australia, Crawley, WA Australia; 7 Department of Neurology, Princess Margaret Hospital, Subiaco, WA Australia; 8 School of Paediatrics and Child Health, The University of Western Australia, Crawley, WA Australia; 9 Department of Anatomical Pathology, PathWest Laboratory Medicine, Queen Elizabeth II Medical Centre, Nedlands, WA Australia; 10 Victorian Clinical Genetics Service, Melbourne, VIC Australia; 11 Department of Endocrinology and Diabetes, Princess Margaret Hospital, GPO Box D 184, Perth, WA Australia

**Keywords:** Disorder of sex development, *Desert hedgehog*, *DHH*, Gonadal dysgenesis, Massively parallel sequencing

## Abstract

**Background:**

*Desert hedgehog* (*DHH*) mutations have been described in only a limited number of individuals with 46, XY disorders of sex development (DSD) presenting as either partial or complete gonadal dysgenesis. Gonadal tumours and peripheral neuropathy have been associated with *DHH* mutations. Herein we report a novel, homozygous mutation of *DHH* identified through a targeted, massively parallel sequencing (MPS) DSD panel, in a patient presenting with partial gonadal dysgenesis. This novel mutation is two amino acids away from a previously described mutation in a patient who presented with complete gonadal dysgenesis. Adding to the complexity of work-up, our patient also expressed gender identity concern.

**Case presentation:**

A 14-year-old, phenotypic female presented with primary amenorrhoea and absent secondary sex characteristics. Investigations revealed elevated gonadotrophins with low oestradiol, testosterone of 0.6 nmol/L and a 46, XY karyotype. Müllerian structures were not seen on pelvic ultrasound or laparoscopically and gonadal biopsies demonstrated dysgenetic testes without neoplasia (partial gonadal dysgenesis). The patient expressed gender identity confusion upon initial notification of investigation findings. Formal psychiatric evaluation excluded gender dysphoria. Genetic analysis was performed using a targeted, MPS DSD panel of 64 diagnostic and 927 research candidate genes. This identified a novel, homozygous mutation in exon 2 of *DHH* (DHH:NM_021044:exon2:c.G491C:p.R164P). With this finding our patient was screened for the possibility of peripheral neuropathy which was not evident clinically nor on investigation. She was commenced on oestrogen for pubertal induction.

**Conclusion:**

The evaluation of patients with DSD is associated with considerable psychological distress. Targeted MPS enables an affordable and efficient method for diagnosis of 46, XY DSD cases. Identifying a genetic diagnosis may inform clinical management and in this case directed screening for peripheral neuropathy. In addition to the structural location of the mutation other interacting factors may influence phenotypic expression in homozygous *DHH* mutations.

## Background

**Fig. 1:**
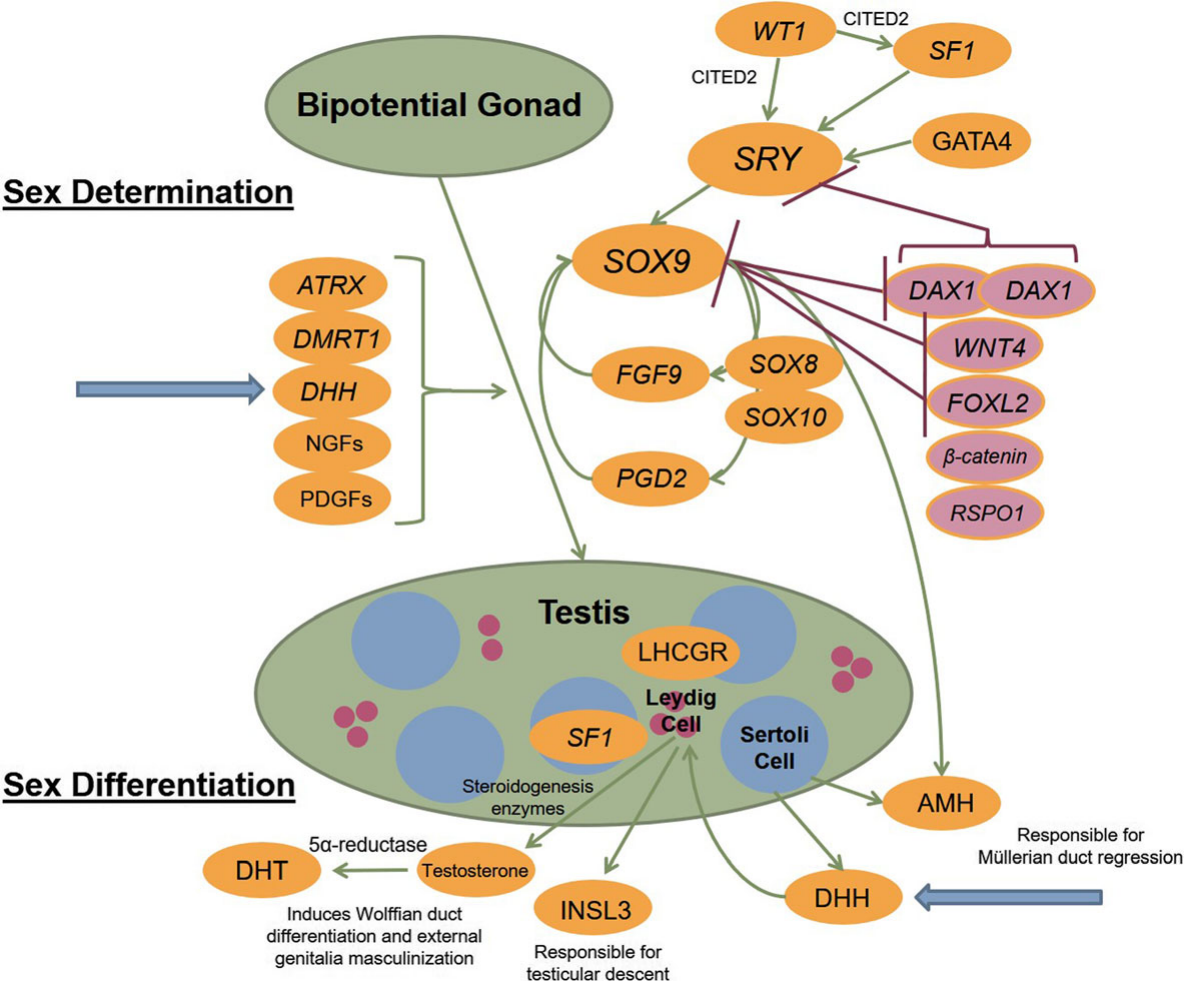
Normal male phenotypic sex development highlighting the role of *DHH*. Testis determination requires *SRY* and one *DAX1* copy, whilst with two *DAX1* copies and a lack of *SRY* an ovary is determined. In the bipotential gonad, *SF1*, *WT1* and *GATA4* upregulate *SRY* which in turn upregulates *SOX9*; *SOX9* is pivotal to testis determination. Upregulation of *SRY* and *SOX9* overcomes the action of genes promoting female sex development including *DAX1* and *β-catenin*. With upregulation of *SOX9,* multiple feed-forward loops then accelerate male pathway commitment. Other genes, including *DHH*, as well as growth factors also have roles in promoting testis determination. Following testis determination, Sertoli cells secrete AMH leading to Müllerian duct regression; Müllerian ducts would otherwise form the fallopian tubes, uterus, cervix and upper vagina. Sertoli cells also secrete DHH which is important for Leydig cell development and Sertoli-peritubular cell interaction. Responding to luteinizing hormone (via LHCGR), a functional Leydig cell produces testosterone via steroidogenesis, requiring SF1 and steroidogenesis enzymes. Converted to DHT via 5α-reductase, testosterone and DHT induce Wolffian duct differentiation (leading to vas deferens, seminal vesicle and prostate formation) and external genitalia masculinization through actions on the androgen receptor. In the absence of functional Leydig cells female external genitalia form and testes fail to descend. Blue block arrows indicate *DHH*’s involvement in male sex development. Lines ending in an arrow denote a positive/up-regulating effect whilst lines ending in a perpendicular bar indicate a negative/down-regulating effect. Abbreviations: AMH– Anti-Müllerian hormone, *ATRX*– *X-linked alpha thalassemia and mental retardation*, *DAX1*– *dosage-sensitive sex reversal, adrenal hypoplasia critical region, on chromosome X, gene 1*, *DHH*– *desert hedgehog*, DHT– dihydrotestosterone, *FGF9*– fibroblast growth factor 9, INSL3– insulin-like protein 3, LHCGR– Luteinizing hormone common G-protein receptor, NGFs– nerve growth factors, PDGFs– platelet derived growth factors, *PGD2*– *prostaglandin D2*, *RSPO1*– *R-spondin-1*, *SF1*– *steroidogenic factor-1*, *SRY*– *sex determining region on the Y chromosome*, *WT1*– *Wilms’ tumour suppressor gene 1*

Male phenotypic development involves testis formation from the bipotential gonad (sex determination) directed by multiple genes that reside on sex and autosomal chromosomes (Fig. 1). Subsequently internal and external genitalia differentiation occurs controlled by factors secreted by the testis (sex differentiation) [1, 2] (Fig. 1). The gene, *desert hedgehog* (*DHH*) plays a role in testis determination [1, 3, 4] and its protein product, produced by Sertoli cells, promotes Leydig cell development by activating Hedgehog signalling [3, 5]. Thus, through this Sertoli-Leydig cell interaction, *DHH* also regulates androgen synthesis and is involved in sex differentiation.

Mutations in the genes involved in testis determination and male sex differentiation, such as *DHH*, cause 46, XY disorders of sex development (DSD). DSD may present in infancy with variable genital ambiguity and 46, XY DSD may present in adolescence with primary amenorrhoea. Historically, a specific diagnosis for 46, XY DSD has remained elusive for many patients. Hormonal testing has limitations with examination of the steroidogenic pathway to determine the adequacy of androgen production and action only yielding a diagnosis in approximately 30% of 46, XY DSD patients [6]. There is normal variation in androgen levels during the neonatal and infantile periods [7] and steroid biosynthesis may change with time in some pathogenic gene variants resulting in misdiagnosis [8]. The use of candidate gene testing for the diagnosis of 46, XY DSD is impeded by the extensive number of genes potentially implicated [9]. Recent advances in sequencing technologies, such as targeted massively parallel sequencing (MPS) of genes in DSD has facilitated molecular diagnosis in up to 43% of 46, XY DSD patients [10]. MPS, allows billions of DNA base-pairs to be sequenced in parallel, yielding substantially more throughput than other technologies, such as Sanger sequencing. Thus, while Sanger sequencing would target a candidate single gene or region, MPS can target all potentially responsible genes.

Herein we describe the clinical course of a patient diagnosed with a novel mutation in *DHH* using the recently described MPS DSD panel [10]. Homozygous mutations in *DHH* have been reported in partial and complete gonadal dysgenesis in only a limited number of 46, XY individuals [11–17]. Co-existence of peripheral neuropathy has been reported in some patients [11, 14–17] as have gonadal tumours [12, 14]. Identification of the genetic aetiology has informed our patient’s management and provides opportunity for genetic counselling for family members. This case was further complicated by gender identity concern. The reported incidence of gender dysphoria in DSD varies from 3.3% in a cohort involving individuals with a range of DSD presentations [18], up to 61.4% in individuals with 5α-reductase deficiency or 17β-hydroxysteroid dehydrogenase type III deficiency [19]. In addition to the underlying DSD, cultural and psychological factors are likely to influence the incidence rate [20].

## Case presentation

A 14-year-old girl was referred for evaluation of primary amenorrhoea and absent pubertal development. She is the oldest of four children born to consanguineous parents (first cousins) of Palestinian background. There were no perinatal issues and there was no past medical history of significance. A maternal sibling had transitioned from female to male as a teen; no further medical information regarding this family member was available.

On examination, our patient presented as a phenotypic female. She measured 154.5 cm in height (10 – 25th centile), weighed 51 kg (50th centile) and was normotensive. There were no dysmorphic features. Cardiovascular, respiratory and abdominal examinations were unremarkable. She had Tanner Stage 1 breast development; pubic hair was Tanner Stage 2. Genital examination demonstrated well-formed labia, a normal vaginal opening and no palpable gonads. There was a prominent clitoro-phallic structure.

Evaluation demonstrated low oestradiol with elevated gonadotrophins (oestradiol 84 pmol/L, follicle stimulating hormone 76 IU/L, luteinizing hormone 37 IU/L); this was confirmed on repeat testing one month later. Testosterone was 0.6 nmol/L. Urea and electrolytes, calcium and fasting glucose were normal. Karyotype revealed 46, XY. On pelvic ultrasound there were no Müllerian structures or gonads identified and there were no concerning mass lesions. On human chorionic gonadotrophin (HCG) stimulation test, baseline testosterone was 1.0 nmol/L and dihydrotestosterone (DHT) 0.2 nmol/L; post HCG testosterone was 0.9 nmol/L and DHT 0.2 nmol/L. Baseline cortisol was 200 nmol/L with cortisol 670 and then 730 nmol/L, 30 and 60 min post 250 mcg of Synacthen.

The diagnosis of a male genotype was difficult for the family. This was further complicated by the diagnosis not initially being disclosed to our patient by her parents. The patient’s mother was concerned about gender identity and assignment especially in view of her sibling’s history. Ultimately our patient was informed of the karyotype result following multidisciplinary review and parental counselling.

**Fig. 2:**
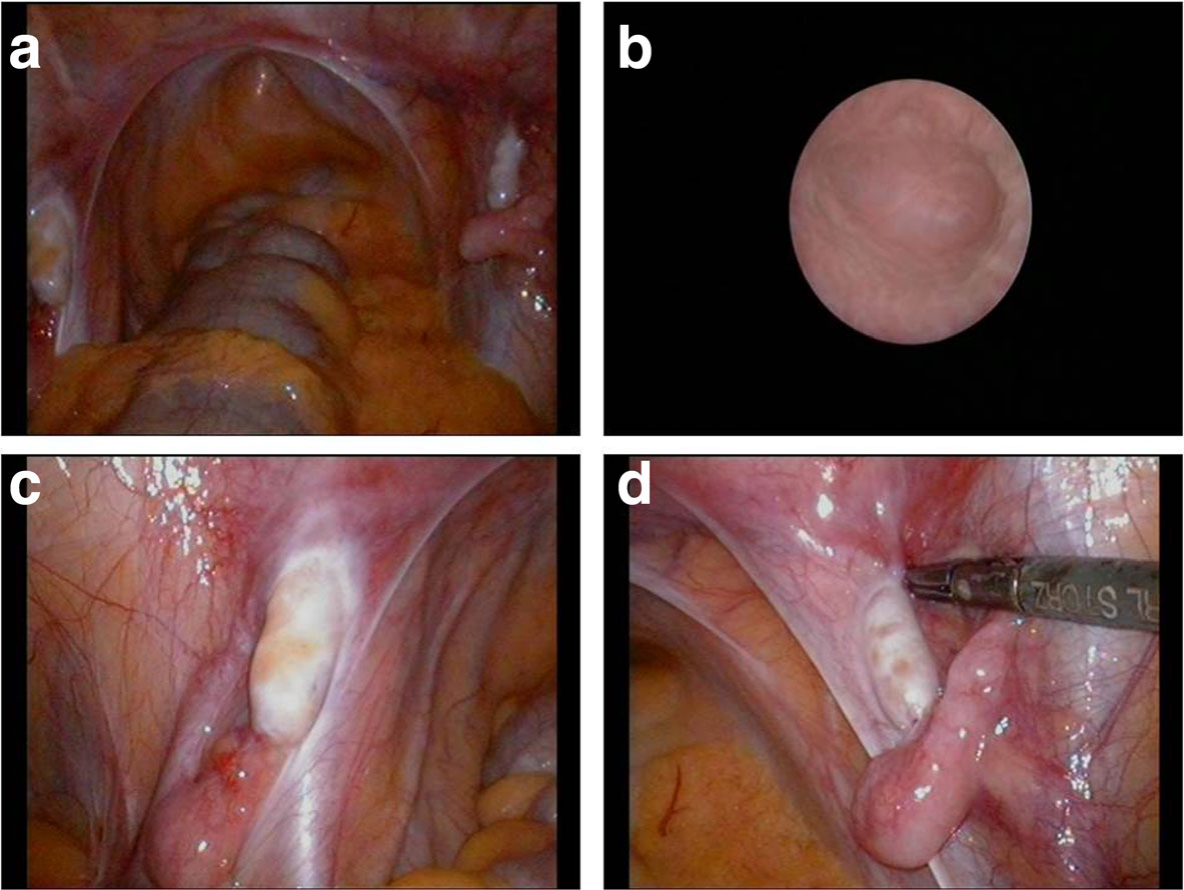
Surgical photos. **a** Laparoscopic view of left and right gonad within the pelvis. **b** Blind ending vagina at vaginoscopy. **c** Left gonad and epididymal flanking structure. **d** Right gonad and epididymal flanking structure

Examination under anaesthesia, laparoscopy, cystovaginoscopy and gonadal biopsies were performed. A blind ending vagina, approximately 6 cm in length from the introitus, was noted. There was no cervix, uterus, fallopian tubes or vasa. There were bilateral abnormal small gonads with a blind ending epididymal structure flanking each (Fig. 2).

**Fig. 3:**
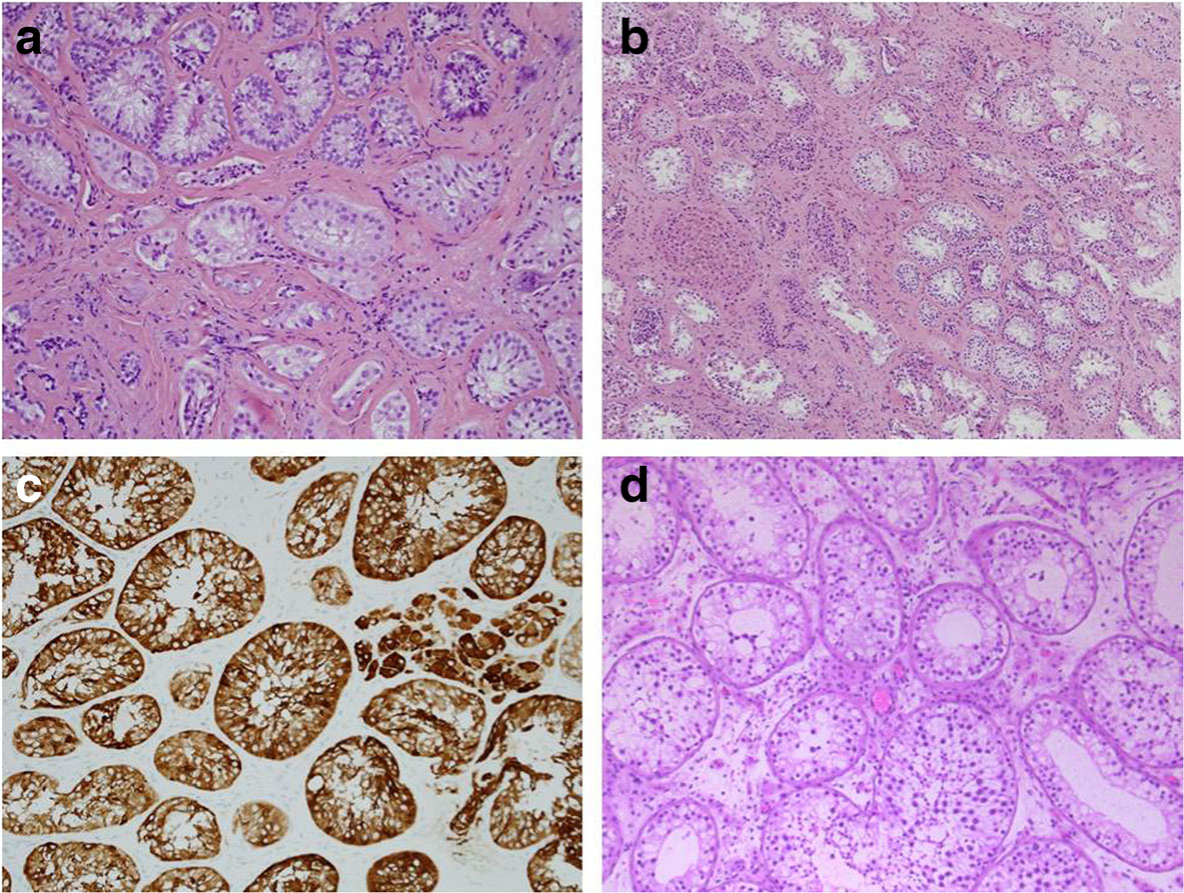
Histopathology. **a** Right gonad excision biopsy, H&E stain, demonstrating seminiferous tubules lined by Sertoli cells without spermatogenesis. There is intervening fibrosis with no definite Leydig cells. **b** Left gonad excision biopsy, H&E stain, demonstrating seminiferous tubules lined by Sertoli cells without spermatogenesis. There is intervening fibrosis with occasional, unusually large clusters of Leydig cells. **c** Left gonad excision, inhibin stain, demonstrating Sertoli and Leydig cell appearance. **d** Normal testis of 15-year-old, showing seminiferous tubules with spermatogenesis

Histopathology from the gonadal biopsies demonstrated bilateral dysgenetic testes with left para-gonadal biopsy showing Müllerian tissue resembling oviduct and the right para-gonadal biopsy showing vaso-epididymal tissue; there were no foci of gonadoblastoma or intratubular germ cell neoplasia. Our patient proceeded to gonadectomy five months later. Histopathology confirmed bilateral dysgenetic testes with no evidence of gonadoblastoma or germ cell neoplasia (Fig. 3).

When reviewing investigation findings, our patient expressed confusion regarding her gender identity stating she always felt that she was a boy. She was referred for psychiatric evaluation. At the time of psychiatric review (four months later), it was noted that our patient had not demonstrated pervasive gender discontent although she periodically expressed thoughts of the cultural advantages of being male. Our patient acknowledged feeling overwhelmed and confused when informed of investigation results. The reviewing psychiatrist concluded that our patient identified as female. Approximately fourteen months following presentation, the patient expressed her wish to proceed with oestrogen replacement therapy.

Our patient had DNA collected under a research protocol examining the molecular genetics of sex determination and gonad development using a MPS targeted DSD gene panel as described by Eggers et al. [10]; consent was obtained from the patient and her mother for this gene analysis. Of the 64 diagnostic and 927 research candidate DSD genes covered in this panel, our patient had a single, rare, non-synonymous variant. This was a novel, homozygous, missense mutation in exon 2 of *DHH* (DHH:NM_021044:exon2:c.G491C:p.R164P). This was confirmed on Sanger sequencing using the primers gccggaataacaaagaatcaac and ggcaacagtactactgcagactc. Our patient’s mother was a heterozygote carrier; other family members have not been tested. This mutation was predicted to be probably damaging by PolyPhen (score 1.0) [21], deleterious by SIFT (score 0.0) [22], and damaging by FATHMM (score − 6.31) [23]. Furthermore, this variant is not present in the ExAC [24], 1000 Genomes Project [25], and NHLBI GO Exome Sequencing Project databases [26], supporting the putative damaging effect of the mutation.

**Fig. 4:**
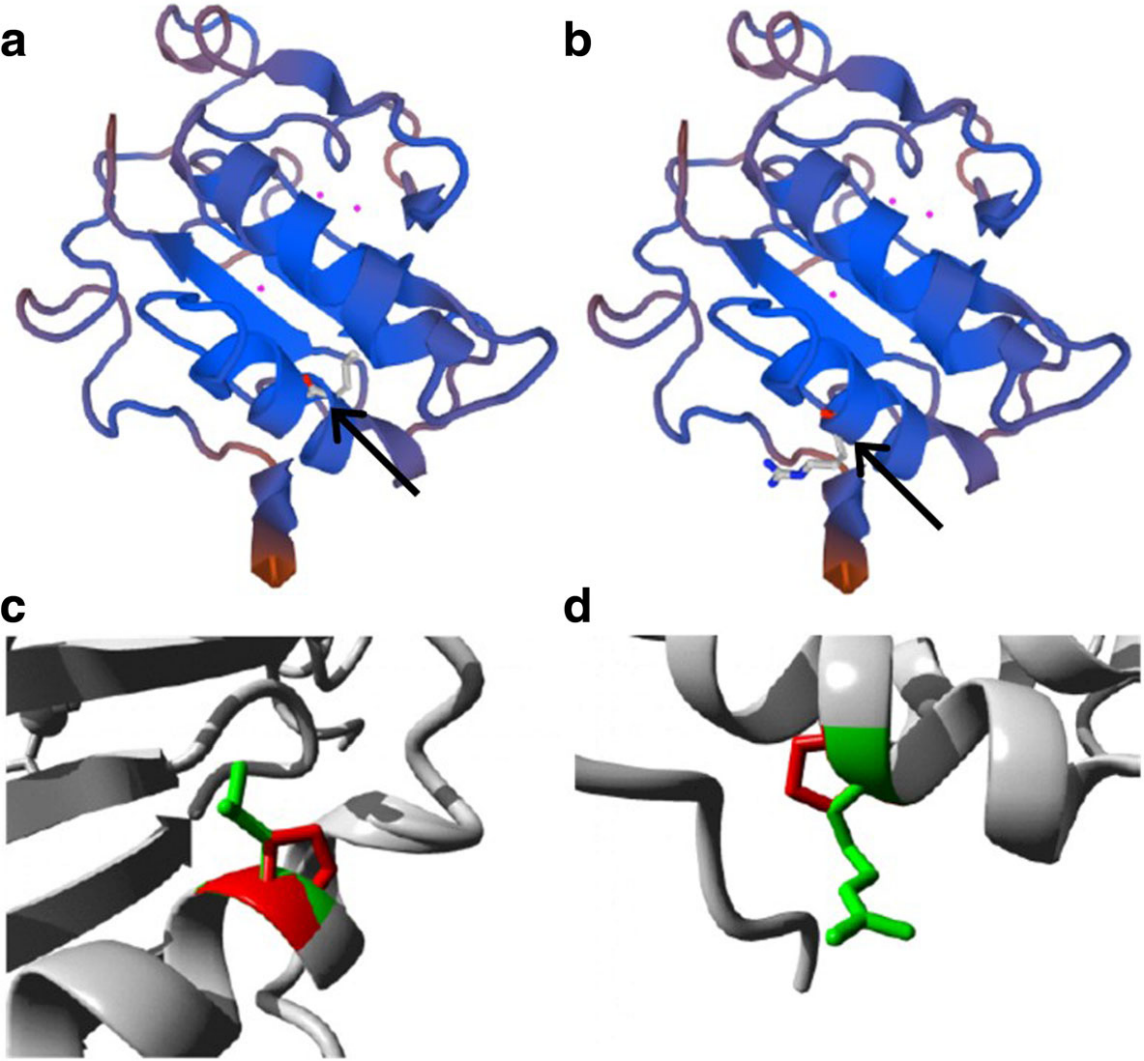
Three dimensional model of DHH. **a** SWISS-MODEL generated 3D model of DHH showing residue Leu162, the position of a previously published mutation [12] and **b** Arg164, the position of our novel mutation. **c** Close-up of the mutation L162P and **d** R164P with the side chains of both the wild-type and mutant residue shown and coloured green and red, respectively. On panels **a** and **b**, black arrows point to position of mutations

We generated a three dimensional protein model of DHH (Fig. 4) using SWISS-MODEL (template ID 3n1g.1.A) [27] and we used HOPE [28] to analyse the structural and functional effects of the mutation. HOPE revealed that the mutated residue is located on a highly conserved position and overlapped three function domains: Hedgehog Protein (InterPro IPR001657), Hedgehog, N-Terminal Signalling Domain (InterPro IPR000320), and Hedgehog Signalling/Dd-Peptidase Zinc-Binding Domain (InterPro IPR009045). The difference in charge and amino acid size between the wild-type and mutant amino acid likely results in loss of interactions with other molecules.

### Follow-up and outcomes

After her psychiatric evaluation, our patient commenced transdermal estrogen in gradually increasing doses. On follow-up out to twelve months post estrogen initiation, breasts had increased in size and were Tanner stage 3. She was pleased with progress of pubertal induction and accepted further escalation in estrogen dose. She no longer expressed gender confusion.

Following the finding of a homozygous *DHH* mutation our patient was specifically evaluated considering the possibility of a peripheral neuropathy. There was no history suggestive of neurological impairment and clinical neurological examination (cranial nerves, gait, co-ordination, tone, power, deep tendon reflexes, touch, vibration and joint position sensation) was normal. Limited nerve conduction studies including left median nerve motor, sensory and F wave response as well as right upper limb somatosensory evoked potential study with stimulation of the median nerve at the wrist were all normal.

Our patient’s family was offered referral for genetic counselling but this has been declined to this point.

## Discussion

Our patient presented with absent puberty and amenorrhoea in the setting of a 46, XY karyotype (46, XY DSD). Although an understanding of male sex development (Fig. 1) and the results of biochemical and anatomical evaluation help narrow the possible diagnoses [29, 30], without gene testing the differential diagnoses in our patient remained broad. In our patient, possible underlying etiologies included the numerable genetic defects causing testicular development disorders (as suggested by Fig. 1), a milder phenotype of steroidogenic factor-1 (SF-1) under-expression, luteinizing hormone receptor defects and homozygous mutation in *DHH* (a disorder of potentially both testicular development and androgen synthesis) [1, 29]*.* In similar scenarios, candidate gene testing provides a molecular diagnosis in only approximately 20% of cases [9]. MPS is more efficient than candidate gene testing (such as Sanger sequencing), its sensitivity allows detection of cases of mosaicism, and using current panels it is more likely to provide a diagnosis [10]. In comparison to whole exome or whole genome sequencing, MPS with a targeted gene panel offers the advantage of shorter processing time with less data handling and analysis without the risk of identifying variants in unrelated areas of the genome (incidental findings) [9, 10]. As the price of MPS technologies decrease, the clinical use of targeted gene panels becomes a better option than Sanger sequencing one or more candidate genes. Where MPS targeted gene panels are not readily available or cost prohibitive, an alternative approach in a suspected autosomal recessive condition (i.e. in the case of consanguineous union) would be microarray and subsequent candidate gene sequencing of potential pathogenic genes within regions of homozygosity.

In our patient, a novel, homozygous *DHH* mutation was detected using the MPS targeted DSD gene panel described by Eggers et al. [10]. This MPS targeted DSD gene panel was conducted as part of a research protocol. DNA from 326 DSD patients has been analysed with this panel [10]; seven new patients with eight novel *DHH* mutations have been identified. Whilst four patients (including our patient) had homozygous or compound heterozygous *DHH* mutations and presented as 46, XY females, three individuals had heterozygous mutations, two of whom were 46, XY undervirilised males. These heterozygous mutations were deemed variants of uncertain significance but suggest *DHH* mutations may explain a broader spectrum of 46, XY DSD. On the other hand, as evidenced by fathers of the reported homozygous *DHH* mutation patients, individuals with a heterozygous *DHH* mutation may be asymptomatic [11]. Functional studies are now being carried out to ascertain the pathogenicity of these variants [Ayers et al. Unpublished]. With these studies and with wider use of MPS methods and analysis of more DSD patients and their relatives, the risks of a heterozygous *DHH* mutation may become clearer.

The *DHH* mutation detected in our patient was a homozygous, missense mutation (exon 2, c.491G > C) in codon 164 (p.R164P). This codon is only 2 amino acids away from a previously published mutation [12]. The mutation in our patient and that previously described both induce a proline mutation in an alpha helix in a highly conserved DHH residue and lead to disruption of the alpha helix [28]. In contrast to our case who had dysgenetic testes and absent Müllerian structures (partial gonadal dysgenesis), the previously published case had bilateral streak gonads with Müllerian structures (complete gonadal dysgenesis). This suggests that in addition to *DHH* mutation localization, other factors may influence phenotype expression. Bitgood et al., noted that in *Dhh*-null male mice bred on different genetic backgrounds spermiogenesis was arrested at different stages. They suggested that other unidentified factors are likely to participate with *Dhh* to express phenotype [31]. This is similar to the *SRY* and *SF-1* genes, where mutations have been associated with a spectrum of 46, XY DSD phenotypes [1, 12].

DHH is a member of the Hedgehog family of signalling proteins which exerts its action through the receptor Patched [32]. Work in mouse models indicates that *DHH* expression is limited primarily to Sertoli cells in the developing testes and to Schwann cells in peripheral nerves [32, 33]. Whilst *Dhh*-null female mice show normal reproduction, *Dhh*-null male mice are sterile [31] with the majority having a feminized appearance [5]. Testes have few Leydig cells, an interstitium filled with fibroblast-like tissue and Sertoli-peritubular cell dysfunction [5], findings similar to that observed in our patient. In nerves, *Dhh*-null mice demonstrate impaired nerve sheath formation with susceptibility to mechanical assault and inflammation-related damage leading to minifascicle formation [32].

A clinical case with features similar to *Dhh*-null mice (46, XY gonadal dysgenesis and minifascicular neuropathy) was first described by Umehara et al. in 1999 [34]. This case was subsequently confirmed to have a homozygous *DHH* mutation [11]. The clinical details of a further eleven individuals with 46, XY karyotype and homozygous *DHH* mutations have been described [11–17] (Table 1). Another two individuals with heterozygous mutations on the background of a 45,X/46, XY karyotype have also been reported [35].

**Table 1:** Previously reported patients with homozygous *DHH* mutations – genetic results and clinical findings

Patient	*DHH* mutation	Presenting age, years	Gonads	Müllerian Structures	Neuropathy
1 [11]	Exon 1, c.2 T > C Initiating codon predicting failure of translation	21	Streak testis and streak gonad	+	+ Age 26 years
2 [12]	Exon 2, c.485 T > C	16	Bilateral streak gonads	+	–
3 [12]	Exon 3, c.1086delG Causes a stop codon, four codons after the deletion.	19	Bilateral streak gonads with bilateral gonadoblastoma	+	–
4 [12]	Exon 3, c.1086delG Causes a stop codon, four codons after the deletion.	26	Bilateral streak gonads with bilateral dysgerminoma	+	–
5 [13]	c.271_273delCAG	26	Bilateral streak gonads	Not reported	–
6 [13]	Exon 1, c.57-60dupAGCC Frameshift mutation resulting in premature termination.	17	Bilateral streak gonads	+	–
7^a^ [14]	Exon 2, c.371G > A	17	Testis (L), Seminoma (R) (age 30 years)	–	+ Early 20s
8^a^ [14]	Exon 2, c.371G > A	17	Bilateral testis with seminoma in situ (R) and gonadoblastoma (age 23 years)	–	+ Age 30 years
9 [15]	c.304-572_492dup	16	Undescended testis in left inguinal canal. Histopathology not reported.	–	+ Age 43 years Associated skin and nail pigmentation and sole skin hyperkeratosis.
10 [15, 16]	c.304-572_492dup	47	Bilateral streak gonads	+	+ Age 39 years
11^b^ [17]	Exon 2, c.519G > T	26	Histopathology unavailable with laparoscopy declined by patient	–	+ Age 26 years Psychomotor retardation and dysmorphic features also reported
12^b^ [17]	Exon 2, c.519G > T	23	Histopathology unavailable with laparoscopy declined by patient	Possible residual structure on ultrasound	–

^a, b^Sisters

Previously reported individuals with 46, XY karyotype and homozygous *DHH* mutations all had female external genitalia and presented with primary amenorrhoea and lack of pubertal development. Internal reproductive anatomy varied with complete gonadal dysgenesis [12, 13, 15, 16] and partial gonadal dysgenesis with [11] or without [14, 15, 17] Müllerian structures described. Cases of complete gonadal dysgenesis tended to have more damaging mutations (failure of translation or premature termination) [12, 13] than the reported sisters with partial gonadal dysgenesis and an associated missense mutation [14], although a case of complete gonadal dysgenesis in a patient with a homozygous missense mutation is reported [12]. Our patient proceeded to gonadectomy due to the increased risk of gonadal tumours with gonadal dygenesis. Gonadal tumours have been reported in *DHH* mutations [12, 14]. Of the 12 described 46, XY homozygous *DHH* mutation cases, six developed a peripheral neuropathy between 20 and 43 years of age [11, 14–17], with no clear genotype-phenotype correlation. It is possible that the cases who did not have evidence of peripheral neuropathy at the time of reporting may still develop symptoms, particularly given that several of these cases were younger than 20 years of age. More recently, a case of minifasicular neuropathy associated with homozygous *DHH* has been reported in a phenotypic female with a 46, XX karyotype [15]. It is only with the diagnosis of *DHH* mutation that we were prompted to consider peripheral neuropathy in our patient. Continued surveillance into adulthood for the potential development of neuropathy will be required.

The psychological distress experienced by our patient and her family as they grasped the nature and implications of her condition added to the complexity of clinical management. Our patient’s parents were initially reluctant to disclose investigation findings to her and upon learning her diagnosis our patient transiently questioned her gender identity. Gender dysphoria is reported in DSD patients, more commonly female patients with prenatal testosterone exposure [20] and conditions associated with virilisation at puberty [19] but recent reports suggest that overall it is uncommon [18, 36]. In contrast, one might expect acute psychological distress for an adolescent or adult receiving a diagnosis of a genotype incongruent to their gender of rearing to be common. Psychological support is advocated for patients with DSD and they are ideally managed in experienced multidisciplinary teams [2, 6].

## Conclusions

In conclusion, our case describes a rare cause of 46, XY DSD presenting in adolescence as primary amenorrhoea and lack of secondary sex characteristic development. Of interest, we report a novel mutation in *DHH* and highlight the potential for psychological distress to develop in the diagnostic work-up of DSD patients. The use of a MPS DSD gene panel enabled the diagnosis of a homozygous mutation in *DHH* and will ensure that our patient continues to receive screening for the potential co-existent peripheral neuropathy associated with *DHH*. We also describe that similar homozygous *DHH* mutations can be associated with variable phenotypes.

## Abbreviations

DHH: desert hedgehog
DHT: dihydrotestosterone
DSD: disorder of sex development
HCG: human chorionic gonadotrophin
MPS: massively parallel sequencing
SF-1: steroidogenic factor-1
